# Epistemic Injustice and Nonmaleficence

**DOI:** 10.1007/s11673-023-10273-4

**Published:** 2023-06-28

**Authors:** Yoann Della Croce

**Affiliations:** Department of Political Science and International Relations, University of Geneva, 40 Boulevard du Pont d’Arve, 1205 Geneva, Switzerland

**Keywords:** Epistemic injustice, Nonmaleficence, Fibromyalgia, Harm

## Abstract

Epistemic injustice has undergone a steady growth in the medical ethics literature throughout the last decade as many ethicists have found it to be a powerful tool for describing and assessing morally problematic situations in healthcare. However, surprisingly scarce attention has been devoted to how epistemic injustice relates to physicians’ professional duties on a conceptual level. I argue that epistemic injustice, specifically testimonial, collides with physicians’ duty of nonmaleficence and should thus be actively fought against in healthcare encounters on the ground of professional conduct. I do so by fleshing out how Fricker’s conception of testimonial injustice conflicts with the duty of nonmaleficence as defined in Beauchamp and Childress on theoretical grounds. From there, I argue that testimonial injustice produces two distinct types of harm, epistemic and non-epistemic. Epistemic harms are harms inflicted by the physician to the patient *qua* knower, whereas non-epistemic harms are inflicted to the patient *qua* patient. This latter case holds serious clinical implications and represent a failure of the process of due care on the part of the physician. I illustrate this through examples taken from the literature on fibromyalgia syndrome and show how testimonial injustice causes wrongful harm to patients, making it maleficent practice. Finally, I conclude on why nonmaleficence as a principle will not be normatively enough to fully address the problem of epistemic injustice in healthcare but nevertheless may serve as a good starting point in attempting to do so.

## Introduction

In her 2007 book *Epistemic Injustice: Power and the Ethics of Knowing*, Miranda Fricker defines epistemic injustice as the wrong done to someone specifically in their capacity as knower (Fricker 2007, 1). She further distinguishes between two kinds of epistemic injustice, respectively those of the testimonial and those of the hermeneutical kind. Whereas testimonial injustice occurs when one suffers a diminished level of credibility imputable to the existence of a negative identity-prejudicial stereotype which undermines one’s testimony, hermeneutical injustice refers to cases when there exists a gap in collective discourse resources which renders one unable to make sense of their social experiences (ibid). The aim of this paper is to focus on epistemic injustices of the testimonial variety as its conceptual nature is constricted by its agential characteristic, making it an ideal philosophical framework in analysing the patient–physician interaction and relationship. This is chiefly the reason why testimonial injustice has been applied to a greater extent than hermeneutical injustice in the medical ethics literature (Harcourt 2021), although there have been some notable exceptions (Carel and Kidd 2014; Kidd and Carel 2017; Kurs and Grinshpoon 2018; Harper 2022).

Since Carel and Kidd’s 2014 article dedicated to mapping the contours of epistemic injustice in healthcare settings (Carel and Kidd 2014), research on this issue has witnessed an impressive growth in the literature. Fricker’s conceptual apparatus has been used to explore and expose epistemic injustices in a wide variety of medical situations and subjects, including for instance psychiatry and mental illness (Lakeman 2010; Sanati and Kyratsous 2015; Kyratsous and Sanati 2016; Carver et al. 2016; Crichton et al. 2017; Scrutton 2017; Gosselin 2018; Newbigging and Ridley 2018; Miller Tate 2019; Bueter 2019; Drozdzowicz 2021), dementia (Spencer 2022), paediatrics (Harcourt 2021; Pham, Storch, and Lazara-Muñoz 2021), ableism and disability (Ho 2011; Scully 2018; Peña-Guzman and Reynolds 2019), medicalization (Wardrope 2014), self-injury (Sullivan 2019), chronic pain and fatigue syndromes (Buchman et al. 2017; Blease et al. 2017; Heggen and Berg 2021), macro allocations of resources (Gallagher et al. 2021), and healthcare practice and patient empowerment at large (Carel and Kidd 2014; Kidd and Carel 2017, 2018; Michaels 2021; Bogaert 2021; Blease et al. 2021; Della Croce et al. 2021; Pot 2022). Epistemic injustice has so far been used as a theoretical apparatus useful in describing and assessing real-world cases that occur in healthcare settings in order to highlight kinds of injustices that have previously been absent from ethical analysis. However, while Fricker’s conceptual toolkit indeed provides medical ethicists with a formidable analytical framework to be used in applied philosophy, little to no work has been undertaken in analysing how epistemic injustice might conceptually interface with principles at the forefront of biomedical ethics and how it might enrich and refine the understanding of the scope and content of physicians’ ethical duties towards patients. As such, I defend the idea that taking epistemic, particularly testimonial, injustice seriously in healthcare has conceptual implications regarding the content of physicians’ ethical duties as healthcare professionals: epistemic injustice is thus more than a phenomenon that surfaces in some medical encounters or healthcare settings, it may also constitute a breach of ethical conduct. In order to demonstrate such a claim, one must first start by showing how epistemic injustice relates with core principles of biomedical ethics. In order to do so, I turn to Beauchamp and Childress’ *Principles of Biomedical Ethics* which, although not without criticism (Clouser and Gert 1990), is by far the most prominent contemporary standard for principlism in medical ethics, and specifically to the ethical principle of nonmaleficence.

A few more words are warranted in order to accurately capture what the added value of exploring the interface of epistemic injustice and the principle of nonmaleficence. It must first be clear that the structure of the argument I present here starts with nonmaleficence and seeks to expand and broaden the scope of the duties covered by the principles through the integration of the framework of epistemic, and as I mentioned specifically testimonial, injustice. Doing so helps shed light on a variety of clinical and moral harms that may not appear to be fully obvious within the scope of the principle of nonmaleficence and its usual entailments. As such, the framework of epistemic injustice helps *specify* the duties of nonmaleficence by enriching the principle with the latest developments in moral theory provided by the framework and its associated grounding in sociological and psychosocial reality. The argument thus resides within the realm of principlism and seeks to enhance it with the tools provided by the framework of epistemic injustice, not replace it.

## Nonmaleficence and Inadvertent Negligence

Beauchamp and Childress list four core principles of biomedical ethics which they argue lay the theoretical ground for the good professional conduct of physicians: beneficence, nonmaleficence, justice, and the respect of autonomy (Beauchamp and Childress 2019). Nonmaleficence, usually considered to be tantamount to the famous ethical maxim *Primum non nocere* (“First/Above all, do no harm”), is the only one of the four principles that prompts physicians not to do something, namely causing harm. While nonmaleficence has had a long history in moral philosophy outside of the realm of medical ethics ranging from Cicero to John Stuart Mill (Bufacchi 2020), its role in contemporary bioethics has been disputed. Although no theorist will disagree regarding whether or not nonmaleficence has a justified spot in the ethical code of conduct of physicians, some have argued that its seemingly hierarchical priority over other principles does not hold ground (Gillion 1985) or that its moral injunctions are best understood as a subset of the principle of beneficence (Frankena 1973). Beauchamp and Childress however argue for a clear analytical separation between beneficence and nonmaleficence, chiefly because as mentioned above nonmaleficence provides physicians with a norm that asks them specifically to refrain from doing *X*, unlike norms of beneficence which demand of physicians to perform actions, such as preventing or removing harm or promote good (Beauchamp and Childress 2019, 157). Specific norms of nonmaleficence include for instance “do not kill,” “do not cause pain or suffering,” “do not incapacitate,” “do not cause offense,” or “do not deprive others of the goods of life.”

A key feature of nonmaleficence is its absence of strict commitment to the notion of intention, in the sense that malevolent intention is not a necessary condition for an action to be qualified as maleficent. As Beauchamp and Childress argue, the scope of obligations of nonmaleficence goes beyond the mere obligation not to inflict harm and also covers the obligation not to impose risks of harm upon patients (ibid, 159). Such obligations imply that physicians, under specific conditions, may be held responsible for maleficent practice even if they did not intend any harm or did not know about the potentiality of any harm being caused. These specific conditions are set by the standard of due care, which simply demands that agents be reasonable and prudent in order to provide appropriate care; or put in other words, which demands that agents not be negligent. Negligence is defined in two ways: first, it may consist in intentionally imposing an unreasonable risk of harm to a patient, or second, in unintentionally but carelessly imposing risks of harm to a patient (ibid, 160). The former is defined as *advertent negligence* whereas the latter is defined as *inadvertent negligence*. I argue that it is in this latter point that an interface with testimonial injustice emerges. Arguing that epistemic injustice of the testimonial kind qualifies as maleficent practice thus calls for the demonstration of two points. First, that testimonial injustice actually results in wrongful harm for patients and second, that testimonial injustice is a consequence of a failure in the standard of due care. I first turn to this latter point and tackle the issue of harm in the next section.

In her account of epistemic injustice, Fricker makes it clear that while there is no direct culprit in cases of hermeneutical injustices, this does not hold true for cases of testimonial injustices (Fricker 2007). As mentioned earlier, testimonial injustices stem from negative identity-prejudicial stereotypes on the part of the hearer which results in an identity-prejudicial credibility deficit for the speaker. Such stereotypes, Fricker takes for example the general statement “women are intuitive,” do not necessarily rest on empirical grounds and are deeply embedded in daily social realities, acting as lenses through which we perceive the social world (Hilton and von Hippel 1996). Physicians, while indeed being trained in medicine and thus rightfully possessing more epistemic legitimacy and authority over medical topics, remain social agents whose beliefs and worldviews have been shaped by their social context and experiences outside of the hospital or medical school grounds. Such reasoning is key to the idea of *multiple agency*: physicians are not only physicians, they are whole persons who happen to also possess the characteristic of being physicians (Sokol 2008). Physicians are thus as prone to reasoning through stereotypes and possessing negative identity-prejudicial stereotypes as non-physicians. In addition to widespread stereotypes based for instance on gender, race, or age, physicians may also display what Kidd and Carel have described as *pathocentric* prejudice, which are prejudices from which epistemic injustices arise towards ill persons, particularly those with chronic somatic conditions (Kidd and Carel 2018). The existence of pathocentric prejudice seems to be reinforced by the idea that physicians appear to hold disease prestige rankings where chronic somatic illnesses such as fibromyalgia have constantly been ranked at the bottom of the list throughout studies (Album and Westin 2008; Album et al. 2017). Pathocentric prejudice hinders the credibility of patients in a number of ways, of which Kidd and Carel highlight two: first that the credibility of patients may be reduced by virtue of them being epistemically incapacitated by their illness, making them confused and epistemically incompetent, and second that their credibility may further be reduced by the perception of hearers that their lives have become dominated by their illness, thus narrowing their focus on their own suffering and losing impartiality and objectivity (Kidd and Carel, 2017). Pathocentric prejudice may be, along with other forms of prejudice, one the vectors of testimonial injustice in healthcare settings. It is crucial to stress that the different forms of prejudice that may cause epistemic injustices are not necessarily exclusive, for they may intersect and reinforce each other should a multiplicity of them be present. As I shall elaborate on later, this is typically the case in fibromyalgia syndrome, where both the illness and the gender of patients (often women) intersect in the production of reduced credibility in healthcare encounters, especially in illness management.

Negative identity-prejudicial stereotypes may operate on a subconscious level, and arguing that the cases of testimonial injustice in medical encounters described in the literature arise from a malevolent intention on the part of physicians would be mistaken. Nevertheless, it is crucial to stress that the lack of intention to deflate a speaker’s credibility does not imply that one’s moral responsibility is to be waived. As Fricker argues, we are responsible for the thorough re-examination of our own stereotypes and biases in order not to let them guide our social interactions without us knowing (Fricker 2007). This proves crucial to linking testimonial injustice with the aforementioned standard of due care, and particularly its condition of inadvertent negligence. Recall that the definition of inadvertent negligence states that in order to be qualified as such, a physician must act in an unintentional but careless way that may impose an unreasonable risk of harm to the patient. Before turning to what exactly is to be understood by “harm” in this definition with regards to testimonial injustice, we must pause and look at the “unintentional” and “careless” features of inadvertent negligence. As I have just shown, it is neither accurate nor justified to accuse physicians of deliberately performing testimonial injustice, that is with the idea of causing harm as a motive. Instead, one may make the reasonable assumption that physicians do not necessarily realize that they are causing harm, largely because they have not proceeded to a thorough re-examination of their stereotypes as Fricker would frame it. This last point however makes the action careless: the harm is caused by an absence of crucial self-reflection that should have taken place in order not to harm the patient. As such, all that is left in order to satisfy the conditions for inadvertent negligence is to show what harm is done to patients when testimonial injustice occurs.

Before turning to what exactly harm entails in such medical encounters, I shall address the problem of beneficence versus nonmaleficence with regards to testimonial injustice. Indeed, a counterargument may be made against mine on the ground that there are cases where a physician ought not to believe a patient for their own good, for instance in the case of a patient experiencing psychosis. While this is indeed true, this also has no bearing on the argument I make here for one simple reason: cases where physicians rightfully dismiss a patient’s testimony or deflate their credibility plainly do not qualify as testimonial injustices. Testimonial injustices occur when one’s credibility is wrongly deflated by virtue of prejudices, and there are many instances in healthcare where credibility may be rightly deflated by virtue of the patient’s mental or physical state at the time of encounter. In addition to these cases, one may also imagine situations where a patient, say for example a drug addict, is rightfully deemed untrustworthy by physicians in interpreting events that are relevant to their medical needs. In other words, testimonial injustice only happens whenever a hearer ought to consider a speaker as a valid epistemic agent but fails to do so (Pohlhaus 2014). My argument is only concerned with cases that effectively qualify as testimonial injustices and therefore where the case for beneficence simply cannot be made. There certainly are plenty of cases where physicians’ epistemic authority is warranted and justified, and attempting to provide a clear cut distinction of when that authority is justified and when it is not would be far too great an endeavour for one paper, if such an endeavour is possible at all. Medicine is and most likely will remain marked by ambiguous situations and moral dilemmas, my point here is solely to argue that there are at least some cases where this ambiguity can be dispelled through careful examination of physicians’ interactions with their patients.

## Harm I: *qua* Knower

Turning to the issue of harm, I shall make a distinction between two distinct, although related, forms of harm that speakers suffer when at the receiving end of testimonial injustice. The first type of harm is done to a person *qua* knower and in this sense does not substantially differ from other cases of testimonial injustices. The second harm, however, is the harm done as a person *qua* patient specifically in healthcare settings and medical encounters. This latter form of harm possesses unique features that especially pertain to medical ethics, especially insofar as its consequences for victims hold clinical implications. In this first section, I focus on the harm done to persons *qua* knowers and shall turn to the problem of harm done to persons *qua* patients in the next.

Before delving into the harm caused by being wronged in one’s capacity of being a knower, it is useful to take a quick detour to what “harm” means in definitions of nonmaleficence. Beauchamp and Childress, drawing direct inspiration from Joel Feinberg, make a clear distinction between two kinds of harm, *normative* and *nonnormative*. Harm in the nonnormative sense implies the setting back or thwarting of one’s interests but does not necessarily involve it being done in a wrongful manner (Feinberg 1984). One can imagine many situations in medical encounters where a physician ought to harm a patient without actually wronging them. Beauchamp and Childress take for example the case of a justified and consented amputation of one’s leg. While such a surgery certainly harms the patient, the harm done is not committed wrongfully (Beauchamp and Childress 2019). On the contrary, harm in the normative sense implies a wrongful setting back or thwarting of one’s interests, where wrongful is defined by an unjustifiable and inexcusable violation of one’s right (Feinberg 1984). The only sort of harm that is of interest here is the latter, nonnormative harms being incompatible with the definition of testimonial injustice for the same aforementioned reasons. If the harm caused by not giving credibility to a speaker was nonnormative, implying that doing so was for the speaker’s own good, then there would be no testimonial injustice to speak of in the first place. As such, it is to be made clear that the harm caused by testimonial injustice is always wrongful, a point that Fricker herself emphasizes (Fricker 2007). A final point that ought to be clarified regarding the relationship between nonmaleficence and harm is that there is nothing in Beauchamp and Childress’s work that seems to indicate that harm necessarily equals physical injury. While many cases of medical malpractice turn out to be ethically problematic on grounds of nonmaleficence (Sharp and Faden 1999; Solomon 2006), this does not imply that all harms that may be committed through maleficence ought to be of physical, tangible nature. There are thus no reasons to *prima facie* dismiss moral or mental harms from the scope of the duty of nonmaleficence.

The harms caused by testimonial injustice are to be classified into two categories, or aspects, which Fricker labels primary and secondary, each of them featuring two sub-categories: the primary aspect of the harm being intrinsic and symbolic, and the secondary aspect being epistemic (or purely epistemic) and practical (Fricker 2007). The primary harm of testimonial injustice intrinsically harms the speaker in their capacity as a knower, or as a valid source of knowledge. Such harm is not trivial. Being wronged in one’s capacity as a knower implies that one is ultimately wronged in a capacity that is crucial to human value. This brings to the conclusion that being wronged in one’s capacity as a knower is tantamount to being wronged in one’s capacity of reason, as such hurting sufferers of testimonial injustices in their very humanity (Fricker 2007, 44; Pohlhaus 2014). While this line of reasoning may seem blatantly Kantian, it is useful to pause here and clarify some of the theoretical underpinnings of the idea of testimonial injustice. Indeed, one may be left to wonder what exactly the added value of the theoretical apparatus of epistemic injustice is should it be a mere declination of Kant’s idea that individuals should always be treated as ends in themselves and never as means in order to respect their autonomy or capacity to self-legislate. It must be made clear that Fricker’s use of this traditionally deontic idea is meant to adequately qualify one of the harms of testimonial injustice, and not made epistemic injustice into a fully-fledged deontic theory. As I shall expand on later, this deontic harm is indeed only one facet of the harms caused by testimonial injustice. Fricker’s framework is indeed broader than a mere application of Kantian principles to the problem she seeks to tackle throughout her work. It is also worth noting that while Fricker herself thinks of her theory as being part of the tradition of virtue ethics, there is some significant disagreement about the extent to which the philosophical foundations of epistemic injustice are truly aretetic or actually deontic (Riggs 2012). While the dispute is yet to be fully resolved, it must be made that clear that even if Fricker’s theory contains elements that pertain to deontology, this does not imply that epistemic injustice is just a simple application of already well-established moral concepts.

Aside from this intrinsic harm, a symbolic harm is also inflicted. As mentioned above, epistemic injustices of the testimonial variety rest on negative identity-prejudicial stereotypes, which are felt by the speaker and reinforced by the hearer, thus adding a layer of social meaning to the injury (Fricker 2007, 44). As for the secondary aspect of the harm, I shall only focus on its epistemic dimension and leave its practical dimension for the next section, since it is of direct relevance to the issues I will be tackling later. Epistemically, a speaker is harmed in the sense that such negative interactions and experiences of injustices and credibility deficit may bring one to lose confidence in their own ability to be a valid source of knowledge. This may happen either through the loss of confidence in one’s beliefs or worse, in their intellectual capacity of forming beliefs (ibid, 46).

It is now useful to return to the short, non-exhaustive lists of moral norms explicated by Beauchamp and Childress in their account of nonmaleficence and see how the three dimensions of harm I have just sketched out conflict with those norms. Of particular relevance are the three following norms “do not cause pain or suffering,” “do not cause offense,” and “do not deprive others of the goods of life” (Beauchamp and Childress 2019, 159). It is fairly straightforward to see how each of these three norms respectively correspond to the primary aspect of the harm in its intrinsic dimension, in its symbolic dimension, and finally to the secondary aspect of harm in its epistemic dimension. Being wronged in one’s capacity of reason is to be wronged in one’s deep sense of humanity, and such can be said to inflict direct suffering upon the person who happens to be on the receiving end of testimonial injustices. Regarding the symbolic dimension of the primary harm, it is here also evident how the layer of social meaning that is experienced by the speaker is to be said to be offensive. Finally, it also appears clear how being able to form beliefs and having faith in one’s self that these beliefs may be true is a crucial part of experience a good life and thus depriving a speaker of this confidence constitutes a deprivation of the goods of life. Obviously, the relationship between those norms and the harms I have described is much more complex than what I have just sketched out here. One could argue that each form of harm described by Fricker corresponds to more than one of the norms of nonmaleficence, instead of the simplified version I propose here, or that different harms may be linked to one single norm. Furthermore, one could wonder what exactly these relationships entail normatively speaking. This calls for a quick clarification that calls back to the very aim of this paper. The importance of the aforementioned relationships simply to provide a *mapping* that highlights the interface between epistemic injustice and nonmaleficence. This step is necessary in order to integrate the moral harms that specifically pertain to the epistemic kind into norms of nonmaleficence. As such, all that is needed for the argument to gain normative traction is to show that these relationships actually exist, thus bringing to the fore a common ground between those two seemingly disconnected philosophical frameworks. It is, in other words, an effort of translation of the moral language of epistemic injustice into the language of nonmaleficence in order to defend the enrichment of the moral duties associated with the latter through the integration of the former’s moral harms.

## Harm II: *qua* Patient

After having examined the set of harms endured by speakers that are victims of testimonial injustice in the general sense, I now turn to harms that have specific consequences that pertain to healthcare, or as mentioned earlier, that have concrete negative implications relevant for clinical practice. Fricker’s final dimension that was left out in the previous section is labelled the *practical* dimension of the secondary aspect of the harm of testimonial injustice. By practical, one must understand the very concrete and pragmatic consequences of having failed to be perceived as a valid source of knowledge: Fricker asks us to think for instance of someone, a black man, who is wrongfully accused of an offense and must thus pay a fine for a crime he did not commit. Despite explaining in detail why he could not realistically be the perpetrator of the offense and claiming that there has been a mistake, the judge simply dismisses his testimony because of a negative prejudice he holds towards black people, thinking of them as pathological liars. The man ends up being charged with a fine for an offense he did not commit. As such, the man effectively lost money (and perhaps his faith in the judicial system): this is the practical dimension of the harm (Fricker 2007). I illustrate this ultimate dimension by using fibromyalgia as a case study that highlights how poor epistemic practices, specifically testimonial injustice, raise ethical issues relevant for both medical and public health ethics.

According to the American College of Rheumatology, fibromyalgia syndrome (or FMS, hereafter referred to simply as fibromyalgia) is a complex and mostly chronic condition whose symptoms most often include muscular pain all over the body, severe fatigue, and tenderness. There exists no test to detect fibromyalgia, its existence being inferred by the absence of other plausible causes for the symptoms (American College of Rheumatology 2019). This has led to controversy amongst the medical community, with some arguing that fibromyalgia is a mind/brain disease and not a musculoskeletal one (Clauw 2014; Bernstein 2016). Furthermore, as is often the case with chronic pain diseases (Buchman et al. 2017), fibromyalgia is more prevalent in women than men, which has been argued to be a reason for frequent dismissal and disbelief during medical encounters (Werner and Malterud 2003). Patients, mostly female, largely report moralizing attitudes on the part of physicians, feelings of guilt, perceptions of misogyny, disbelief, and accusations of malingering throughout most of their medical encounters (Quintner 2020). This in turn has led some to argue that fibromyalgia is a prime example of epistemic injustice in healthcare, especially since evidence-based practice proves to be significantly deficient in its management and understanding (Heggen and Berg 2021). Testimonial injustice displays itself through different dimensions in the case of fibromyalgia, namely through the diagnosis and the management of the condition. The first dimension (meaning the difficulty to get an appropriate diagnosis because of negative stereotypes affecting the ill person, which are mostly female as mentioned above) has become less of a problem because of the now clearer criteria of diagnosis, thus leading physicians to be more familiar with the condition and better informed about its characteristics. The second dimension, referring to the management of the illness itself, is where the heart of the issue currently lies, and what drives the clinical harms that go beyond the moral wrongs of epistemic injustice.

One may wonder, in the case of injustice that affects the management of the illness, on exactly what negative stereotype the injustice operates. The response to this question is manifold. As I have argued, both pathocentric prejudice (as one can infer from the abysmal ranking of fibromyalgia on the disease prestige scale) and prejudice towards women are at play here. It is a possibility that negative stereotypes about women have a causal role in the emergence of the pathocentric prejudice regarding fibromyalgia, whether this is actually the case or not is however largely beyond the scope of this paper. In order to support the case I wish to make here, all that is needed is however to simply acknowledge that these two forms of prejudices exist and intersect in the management and understanding of fibromyalgia within the physician–patient relationship.

Systematic disbelief and dismissal, both clear outputs of testimonial injustice, are indeed not only harmful in themselves, for they bring along some dire consequences with them. The range of consequences include over/under-medication and increased risks of drug abuse (Hayes et al. 2010; Durif-Bruckert et al. 2014), loss of confidence in healthcare and subsequent loss of adherence to treatments (Dobkin et al. 2003; Dobkin et al. 2006), and increased stress due to repeated gaslighting, which in turns worsens the condition itself (Raymond and Brown 2000). All of these dimensions belong to Fricker’s practical dimension of the harm of testimonial injustice, but it is clear that these negative consequences should also be used as a marker that reveals something beyond the pain endured by the patient/speaker, that is the failure in physicians’ moral obligations that have led to this state of affairs. The picture looks even grimmer when one realizes that these consequences reinforce the larger trend of chronic illnesses being heavily linked to significant unwarranted disadvantages in both quality and duration of life (Stutzin Donoso 2018).

## Nonmaleficence, Beneficence, and Achieving Epistemic Justice

I have so far fleshed out the conceptual links between epistemic injustice of the testimonial kind and the principle of nonmaleficence, as well as given an illustration of how epistemic injustice not only morally harms the person against whom the injustice is committed against but also potentially negatively impacts their medical condition, partly due to the hindrance of the physician–patient relationship. Thus, while those conceptual links may now appear clearer, I have said little about what ought to be done in order to limit, or at best rectify, the damage of epistemic injustice in medical encounters. This proves to be a trickier endeavour than what it may seem at first glance. Recall that nonmaleficence, in contrast with beneficence, requires that physicians refrain from performing a certain class of actions which wrongfully harm their patients, whereas beneficence demands of physicians to perform actions which promote the well-being of patients. As such, defining epistemic injustice as a maleficent act, meaning that it wrongfully harms the patient, makes it logically fall under the principle of nonmaleficence when ascribing moral responsibility and circumscribing the normative duties of physicians. Therefore, all that can be normatively argued for on such grounds is for physicians to simply refrain from committing epistemic injustice in their medical practice. Such a conclusion is however only mildly satisfactory, for it fails to fix any substantial issue. Since epistemic injustices, particularly testimonial, rest on the existence of negative identity-based prejudices, and since prejudices find their origins in stereotypes that exist beyond the mere collection of individual opinions, it is simply mistaken to believe that prejudices will disappear by increasing awareness of the concept of epistemic injustice amongst physicians. While this is certainly a necessary step to undertake, it certainly will not be a sufficient one. What is needed is not only the correction of epistemic injustice but also the promotion of epistemic justice.

How to best promote epistemic justice has been the subject of debate, and so far little to no consensus exists in the contemporary literature. A promising approach can be found in using the tools of restorative justice, through the acknowledgement of the injustice, the amendment of the hearer’s view in accordance with that of the speaker, both of which should hopefully lead to forgiveness and the rebuilding of trust in the epistemic relationship (Almassi 2018). It is however unclear how the principle of nonmaleficence gives sufficient normative traction to ask physicians to undertake these steps. The principle of beneficence, or the principle of justice to the extent that epistemic rights and privileges are goods that ought to be distributed fairly and equally (Miller and Pinto 2022), seem far better equipped to tackle the problem of promoting epistemic justice in healthcare. This, however, is beyond the scope of this paper and its conceptual goals. While nonmaleficence may not be a suitable candidate for securing epistemic justice in physician–patient relationships, it may still provide us with a good starting point in doing so, even if the work ought to be finished with the help of other principles. A good place to begin trying to limit epistemic injustice would be for instance to familiarize medical students with the theoretical apparatus of epistemic injustice, which has for now been given little attention outside of the realm of moral and social philosophy. Doing so would help to-be physicians better understand the complex power dynamics at play between them and their patients in clinical encounters, largely through the acknowledgement of how their position in society and their worldviews are not only socially contingent, but also affect the way they approach their relationships with patients. Doing so encourages self-reflection and critical examination, both of which have been argued to be crucial in achieving epistemic justice in psychiatry for example (Leblanc and Kinsella 2016). This is furthermore consistent with recent advances in the philosophy of medicine, which makes salient the idea of understanding the physician not only *qua* specialist but also *qua* person, embedded in societal norms and practices which they both shape and are shaped by (Marcum 2017). Before seeking to correct epistemic injustice, it is first necessary to be able to locate it in medical practice, something that cannot be done if physicians are not aware of the concept and its subsequent harms. As such, emphasis during medical training or through professional certifications, in conjunction with institutional will to tackle the issue provides a good starting point in doing so. While this certainly will not be enough to ensure epistemic justice and guarantee trustful relationships between physicians and patients, it is nevertheless a good place to begin. It is however crucial to highlight that while epistemic injustice as maleficence and epistemic justice as beneficence are distinguishable, and must be distinguished, from a conceptual point of view, they are in practice inextricably intertwined. The first is after all a necessary condition for the achievement of the second: there will be no justice as long as injustice goes unnoticed. Establishing precisely what epistemic justice requires and how it is tied to beneficence is however an endeavour that ought to be undertaken separately from what I have attempted to achieve here. Since the reduction of injustice is a precondition for the realization of justice, it was nevertheless crucial to first shed the light on the kind of moral and clinical wrong I have sketched out in the previous pages, before delving further into the exact demands of justice when it comes to epistemically disadvantaged patients.

## Conclusion

I have argued that in the context of medical ethics, epistemic injustice can be more than an analytical framework that helps us disentangle ethical conundrums or shed light on morally problematic interactions that were previously invisible. Epistemic injustice, and specifically testimonial, may also be used on the conceptual level to better our understanding of the moral obligations of physicians, particularly that of doing no harm. As I have argued, it appears clear that committing testimonial injustice as a physician during medical encounters can be safely said to be harmful towards the patient, be it in their dignity, confidence in themselves, social identity, or even in their illness. As such, this paper is a call for two things. First, for medical ethicists to further examine the normative implications that epistemic injustice holds for medical ethics and professional codes of conduct, and second for physicians to be better equipped, trained, and sensitized to these dimensions of injustice in their clinical practice. Indeed, while maleficence is usually thought to pertain to physical harm, some of the most pervasive, silent, and destructive harm a physician can inadvertently do may operate on a level invisible to the eye or X-ray scans. Nonmaleficence may not provide fully sufficient normative traction in order to promote epistemic justice, but it provides physicians and medical ethicists with a good place to start in addressing the issue on both a conceptual and deontological level.
